# Additive manufacturing and rheological characterization of ceramic matrix composite inks with high fiber volume loadings

**DOI:** 10.1557/s43579-025-00780-3

**Published:** 2025-07-31

**Authors:** Mitchell R. Donoughue, Joshua D. Anderson, Dev I. Thawani, Jean Corraliza-Rodriguez, Anna Mathis, Monique S. McClain

**Affiliations:** 1 School of Mechanical Engineering, Purdue University, 610 Purdue Mall, West Lafayette, IN 47907 USA; 2 School of Aeronautics and Astronautics, Purdue University, 610 Purdue Mall, West Lafayette, IN 47907 USA; 3 Department of Mechanical and Aerospace Engineering, University of Central Florida, 12760 Pegasus Drive, Orlando, FL 32816 USA

**Keywords:** 3D printing, Additive manufacturing, Aerospace, Ceramic, Fiber, Composite

## Abstract

Additive manufacturing of fiber-filled ceramic matrix composites (CMCs) can be used to tune local properties *via* controlled fiber orientation. Increasing the loading of fibers in additively manufactured CMCs is needed to improve the fracture toughness, yet printing CMCs with high fiber loadings (> 20 vol%) remains challenging. In this work, the combination of viscous silicon oxycarbide preceramic resins, short carbon fibers (50 µm × 7 µm), and Vibration-Assisted Printing enable printing of a mixture with 39.4-vol.% carbon fiber (total loading of 43.3 vol%) with omnidirectional fibers within the bead.

**Graphical abstract:**

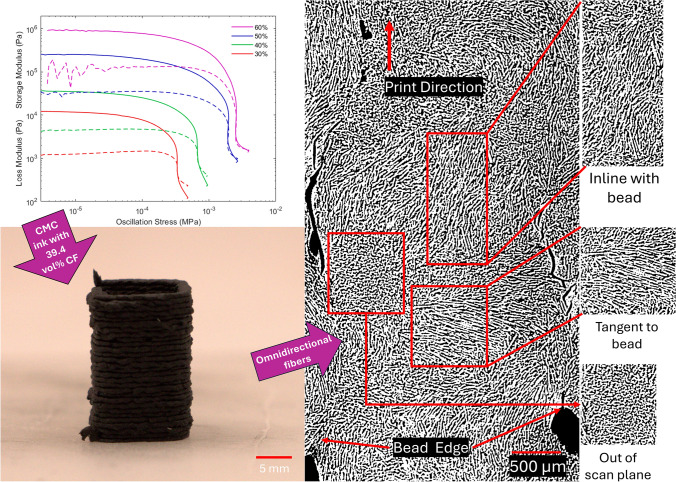

**Supplementary Information:**

The online version contains supplementary material available at 10.1557/s43579-025-00780-3.

## Introduction

Additive manufacturing (AM) of ceramic matrix composites (CMCs) offers the promise of producing strong, high-temperature materials with complex geometries rapidly and with significantly reduced tooling costs.^[1]^ CMCs with fiber reinforcement is an active area of research because fiber inclusion can improve properties, such as fracture toughness^[2]^ and wear resistance.^[3]^ In traditional manufacturing methods, 40–65 vol% of continuous fibers is commonly used to improve fracture toughness.^[4]^ Although continuous fibers are generally preferred due to their load-bearing ability, the majority of material extrusion AM fiber-reinforced CMC work will continue to focus on the integration of cheap whisker fibers until more AM techniques are capable of integrating a substantial amount of continuous fibers into prints.^[5,6]^

Despite the benefits of increasing the fiber loading, there are challenges in increasing the volume loading of fibers over 20 vol% for AM.^[1,7]^ Notably, fibers can entangle, which has been shown *in situ* at low loadings of silicon carbide (SiC) fibers (10 wt% or 5.75 vol%).^[8]^ Furthermore, a higher yield stress means that it will be harder to initiate the flow for printing.^[9]^ One strategy to produce CMCs with improved functionality is to accept low fiber loadings (e.g., 10 vol%), but to supplement that with high ceramic particle content (e.g., 47.5 vol%).^[10]^ Other work has shown success using solvents to extrude preceramic polymers with fiber volume loadings up to 33 vol% through 837-µm diameter nozzles, although this mixture was not used for printing and porosity is a common symptom of solvents.^[11]^ Shorter fibers (50 µm) also tend to improve extrusion properties.^[12]^ Furthermore, AM techniques such as Vibration-Assisted Printing, VAP^[13]^ are able to extrude more viscous mixtures at higher speeds and resolutions than standard DIW processes.^[14]^ VAP has also been used to 3D print CMC mixtures with longer fibers (100 µm) up to 45 wt%.^[15]^

Despite these advancements, mixtures with a high volume loading of fibers typically do not produce the desirable rheological properties required for material extrusion AM techniques, such as Fused Filament Fabrication (FFF) or Direct Ink Writing (DIW). It has been shown that printing polymers with high fiber loadings over 20 vol% can be exceptionally challenging.^[7,16]^ For example, doubling carbon fiber content from 20 to 40 wt% can triple viscosity^[16]^ and make the pressure drop across an extruder system higher than the limit required for successful extrusion. Furthermore, concentrated suspensions can exhibit shear thickening and higher aspect ratio particles have higher viscosities.^[17]^ However, it is noted that higher equilibrium moduli and shear stresses are desirable to form free standing structures, which typically increases with fiber loading.^[12]^ A combination of print strategy and new AM techniques are required to 3D print non-ideal, highly filled CMC inks to increase the range of functionality that can be obtained.

Furthermore, material extrusion AM techniques orient the fibers along the tool path due to shear alignment^[18]^ and fiber orientation is critical for controlling mechanical properties,^[19]^ thermal conductivity, and electrical properties, to name a few.^[20]^ Although high alignment can promote anisotropic properties, random fiber orientation within a print could offer opportunities for improved isotropy. For example, randomly oriented fibers in CMCs have been shown to lower the coefficient of friction and volume of wear, which is desirable for bearings.^[3]^

The main objective of this work is to offer a path to 3D print mixtures with a high loading (> 30 vol%) of fibers. We do this by manufacturing a complex ink with almost 40 vol% of fibers. We conduct line print tests to determine the effect of rest time between extrusions and first layer height on extrusion quality using optical profilometry. We then use this to inform our strategy to 3D print free standing structures using a highly loaded ink. Finally, we report the unique local fiber orientation within the print. This work enables a path forward to higher fiber volume loading AM of CMCs, or other similar fiber-filled polymers, for a wide range of applications.

## Materials and methods

### Ink preparation

The inks were prepared using SPR-688 resin (a polysiloxane-based resin from Starfire Systems), which has a measured viscosity of approximately 1000 cP (1 Pa s).^[21]^ A resin with a higher viscosity was chosen to reduce separation from fibers during the print process. The loading of carbon black (20–50 nm, Vulcan XC-72R) and fumed silica (< 0.02% 325 Mesh Residue, Sigma–Aldrich, S5130) were both fixed at 2.5 wt% for all mixtures (5 wt% or 3.9 vol% total). In addition to this, the main filler was carbon fiber (K223HM, 11 µm diameter, 50 µm length, Mitsubishi Chemical Corporation) which was included at loadings of 25 (14.8), 35 (22.0), 45 (30.0), or 55 (39.4) wt% (vol%). These mixtures are labeled as 30, 40, 50, and 60% throughout the rest of the study and refer to the total wt% of fibers, fumed silica, and carbon black. Carbon fiber was weighed using an Ohaus Pioneer microbalance (measured to 0.001-g accuracy). All other ingredients were weighed using a Torbal AGCN220 microbalance (measured to 0.001-g accuracy).

The SPR-688 resin was stored in a refrigerator at 5°C. During preparation of the ink formulation, the resin was used immediately without equilibration to room temperature. All other ingredients were stored at room temperature and used without further processing. Then, 10-g mixtures were made in 1 oz. thick wall mixing containers (Pt. # 67WT48, Cary Company). Mixing was performed in a FlackTek (DAC 515-200 Pro) for 30 s at 2000 RPM. A spatula was then used to scrape the sides and bottoms of the container to integrate any remaining resin or solids. This procedure of mechanical and hand mixing was repeated until mechanical mixing had been performed at least twice and the mixture was visually homogeneous.

### Rheology experiments

Rheological measurements were performed on a TA Instruments Discovery HR-30 with an environmental test chamber. To maintain a consistent temperature, the chamber was set to 28°C for all testing. To minimize wall slip, 25-mm crosshatched parallel plates were employed. The mixtures were loaded between the plates and then trimmed at a gap of 550 µm. A gap of 500 µm was used for all testing to minimize ink squeeze out while maintaining an adequate separation for the carbon fiber inks to be employed. Before testing, the inks were preconditioned using a shear rate of 0.01 s^−1^ and then left to equilibrate in the rheometer for 1200 s at 28°C with no applied stress. The following procedures were performed to evaluate the rheology of the ink: a ramp loop, a constant shear rate, and an amplitude oscillation test.

Ramp loop procedures used shear rates from 0.01 to 4 s^−1^ to evaluate time-dependent non-Newtonian behavior. While ramps to higher shear rates were attempted, these ramps often resulted in the material exiting, or squeezing out, of the gap. A maximum shear rate of 4 s^−1^ resulted in more consistent measurements. Ramp loops were analyzed for thixotropic or rheopectic behavior by observing the relative position of the ramp-up and ramp-down curves. Time-dependent rheology behavior was also assessed using a constant shear rate test which measured the viscosity as a function of time at a shear rate of 0.0001 s^−1^.

Oscillatory amplitudes were characterized using strains between 0.0001 and 160%. The critical stress and strain were observed where the storage and loss moduli are equal [tan(*δ*) = 1]. The equilibrium storage modulus, *G*′_o_, was also determined using the modulus at the lowest shear rate with consistent (i.e., minimal noise) data for amplitude oscillation.

### Printing

After mixing, the ink was loaded into a 10 cm^3^ syringe (item no. 8001042, Fisnar Quanx). The syringes were then capped and mixed at 2000 RPM for 30 s in the FlackTek. Only the 60% (43.3-vol% total solids, 39.4-vol% carbon fiber) ink was printed. An 18-ga nozzle (837 µm inner diameter) was used for line, zig-zag, and rest time print tests. Printing was performed on an Ender 5 Plus printer that was modified with Vibration-Assisted Printing,^[13]^ which has been used to extrude similar mixtures at lower fiber loadings in the past.^[15]^ Slicing was performed with the spiral vase option in SuperSlicer to improve printability and print quality. The print speed and extrusion multiplier were changed until the resulting print bead was as uniform as possible. The final extrusion multiplier was 1 and the speed was 1 mm/s for print tests.

Using these print parameters to perform line and zig-zag (45° angle) tests, three layer heights (0.6 mm, 0.9 mm, and 1.2 mm) were compared for line consistency and turning performance. Furthermore, the effect of rest time, or the time when the syringe plunger was not moving and exerting force on the ink, on the print quality was quantified using rest times of 1, 2.5, 5, 10, 20, and 40 s between each line.

Taller, free standing square single-walled towers were printed using a layer height of 0.9 mm and no rest time (continuous printing). The height of the towers was 15 mm and the width/length was 10 mm. All prints were scanned using a Keyence VR-6000 optical profilometer for analysis. Line tests were analyzed using the Keyence VR-6000 software to determine the bead arithmetic mean surface roughness along the centerline. Zig-zag tests were analyzed to compare the bead width between the beginning and end of the test. The rest time tests were analyzed along the centerline to determine the degree of flow instabilities present. One tower was scanned immediately after printing and 8 days after printing to determine the change in height.

To determine the fiber alignment within a bead, a structure was analyzed using X-ray micro-computed tomography (mCT). One rectangular prism, 10 mm × 10 mm × 2 mm, was printed using the same settings as the single-walled towers. The ink formulation was changed slightly by substituting 0.4 wt% of the mixture with a platinum catalyst (CAT 776, Starfire Systems) to allow curing. This prism was cured at 200°C for 12 h. A Phoenix V|tome|x M300 was run at 50 kV and 275 µA with no prefilter. The detector had a dwell time of 500 ms and a voxel resolution of 2.25 µm.

## Results and discussion

### Rheology measurements

**Figure 1:**
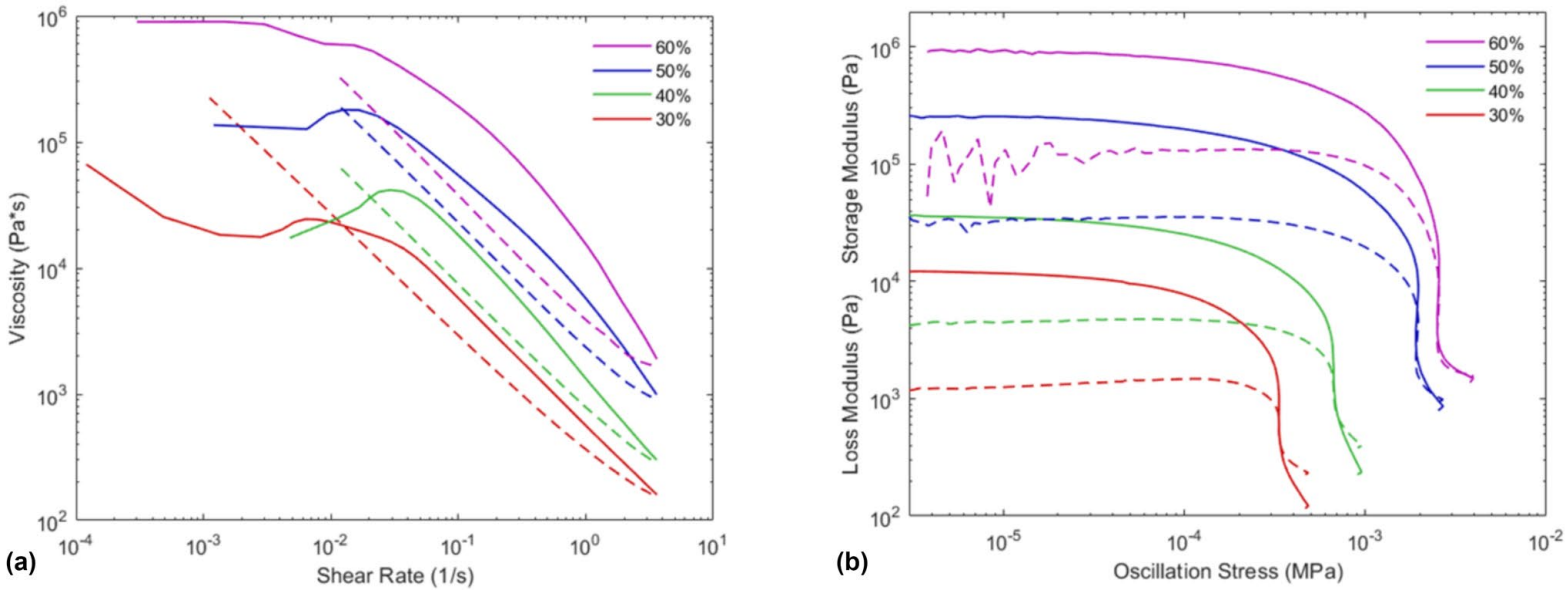
Ramp loop and oscillatory rheology curves of the 30, 40, 50, and 60% inks. Ramp loops (a) show the ramp up in solid lines and ramp down shown in dashed lines. Amplitude oscillation curves (b) are shown with solid lines for the storage modulus and dashed lines for the loss modulus. A yield point was observed in all mixtures.

Ramp loop experiments showed non-Newtonian behavior across all tested mixtures, as seen in Fig. 1(a). The solid lines show the ramp-up behavior and the dashed lines show the ramp down behavior. In general, the measured viscosities increase by approximately half a decade per 10-wt% solids loading increase. At shear rates below 0.1 s^−1^, the ink behavior is more complex. The 30% ink shows shear thinning on the ramp up from 0.0001 s^−1^ until it reaches a local minimum around 0.003 s^−1^. Then, the ink shows shear thickening behavior from this local minimum until it reaches a local maximum in viscosity around 0.006 s^−1^. Finally, the ink shows linear shear thinning again from 0.006 s^−1^ and throughout the ramp down. This mixed shear thinning and thickening behavior is observed elsewhere in literature, including in carbon fiber systems.^[12]^

The 40 and 50% inks both show shear thickening at low shear rates during ramp up until they reach a local maximum around 0.029 and 0.014 s^−1^, respectively. Both the 40 and 50% inks then show relatively linear shear thinning beyond this maximum and throughout the ramp down. Finally, the 60% ink shows an initial shear thinning region during ramp up and hits a small plateau between 0.009 and 0.015 s^−1^. The 60% ink shows shear thinning above shear rates of 0.015 s^−1^ during the ramp up and throughout the ramp down.

The time-dependent viscosity behavior is evident when comparing the ramp-down and ramp-up curves. Both the 30% and 40% mixtures show crossover points between the ramp-up and ramp-down curves at shear rates of approximately 0.01 s^−1^ and 0.02 s^−1^, respectively. At lower shear rates, they both showed rheopectic behavior, where the ramp-down curve is higher than the ramp-up curve. At higher shear rates, they transitioned to thixotropic behavior, where the ramp-down curve is lower than the ramp-up curve. The viscosities of the ramp-up and -down curves were equal around 0.01 s^−1^ for the 50% mixture, indicating a likely transition to rheopectic behavior, but is not conclusive. No crossover was measured for the 60% mixture. All inks showed thixotropic behavior at higher shear rates (> 0.03 s^−1^), although the hysteresis is more pronounced at higher loadings. Similar transitions between rheopecty and thixotropy are observed in literature.^[22]^

The transition between rheopectic and thixotropic behavior, as well as transitions between shear thickening and shear thinning behavior, at different shear rates is certainly less than ideal for a printable ink. For example, starting or restarting flow from rest (i.e., low shear rates) may be difficult to overcome due to the shear thickening behavior at low shear rates. Once the non-ideal low shear rate behavior is overcome, the inks show controllable shear thinning behavior. The high viscosity around 0.001 s^−1^ for the 60% mixture in Fig. 1(a) likely contributes to printing difficulties, as this plateau must be overcome for consistent extrusion. These difficulties are particularly difficult to mitigate, given the high viscosity of the mixture. The data from the constant shear rate test and the complex viscosity data are shown in the supplemental information, but support the time-dependent behavior and high viscosity of the ink.

Amplitude oscillation tests showed a yield stress for all tested mixtures as seen in Fig. 1(b). Yield stress and strains are outlined in Table I. The yield stress observed in both the 50 and 60% inks is higher than the yield stresses observed in comparable lower fiber loading inks in literature.^[12]^ The large yield stress in the 50 and 60% ink should allow printing of taller structures with minimal slump, although it is noted that it will be harder to start the flow.^[23,24]^

**Table I:** Yield stress, strain, and equilibrium storage modulus from oscillatory amplitude sweeps.

Mixture (wt% solids)	Yield strain (%)	Yield stress (kPa)	Equilibrium storage modulus (kPa)
30	59.1	0.34	12
40	72.8	0.69	37
50	77.2	1.36	255
60	147.4	1.65	911

Amplitude oscillation tests also showed significant noise in the loss modulus at stresses < 10^−5^ MPa for the 60% mixture. This noise is attributed to the instrument having difficulty assessing high viscosity mixtures at low strain rates (below 0.01%).^[25]^ While the noise makes visual confirmation of the linear viscoelastic region (LVR) difficult, the stress–strain curve was linear for the 60% mixture at strains below 0.001 s^−1^.

The equilibrium storage moduli (*G*′_o_) for all inks are also shown in Table I. *G*′_o_ increases with solids loading and increases sharply for mixtures > 40%, indicating a significant change in rheological properties. As *G*′_o_ influences properties like gap spanning^[26]^ and buckling at higher layers,^[23]^ this increase in *G*′_o_ will aid in the creation of taller and more complex structures.

### Line and zig-zag print quality

**Figure 2:**
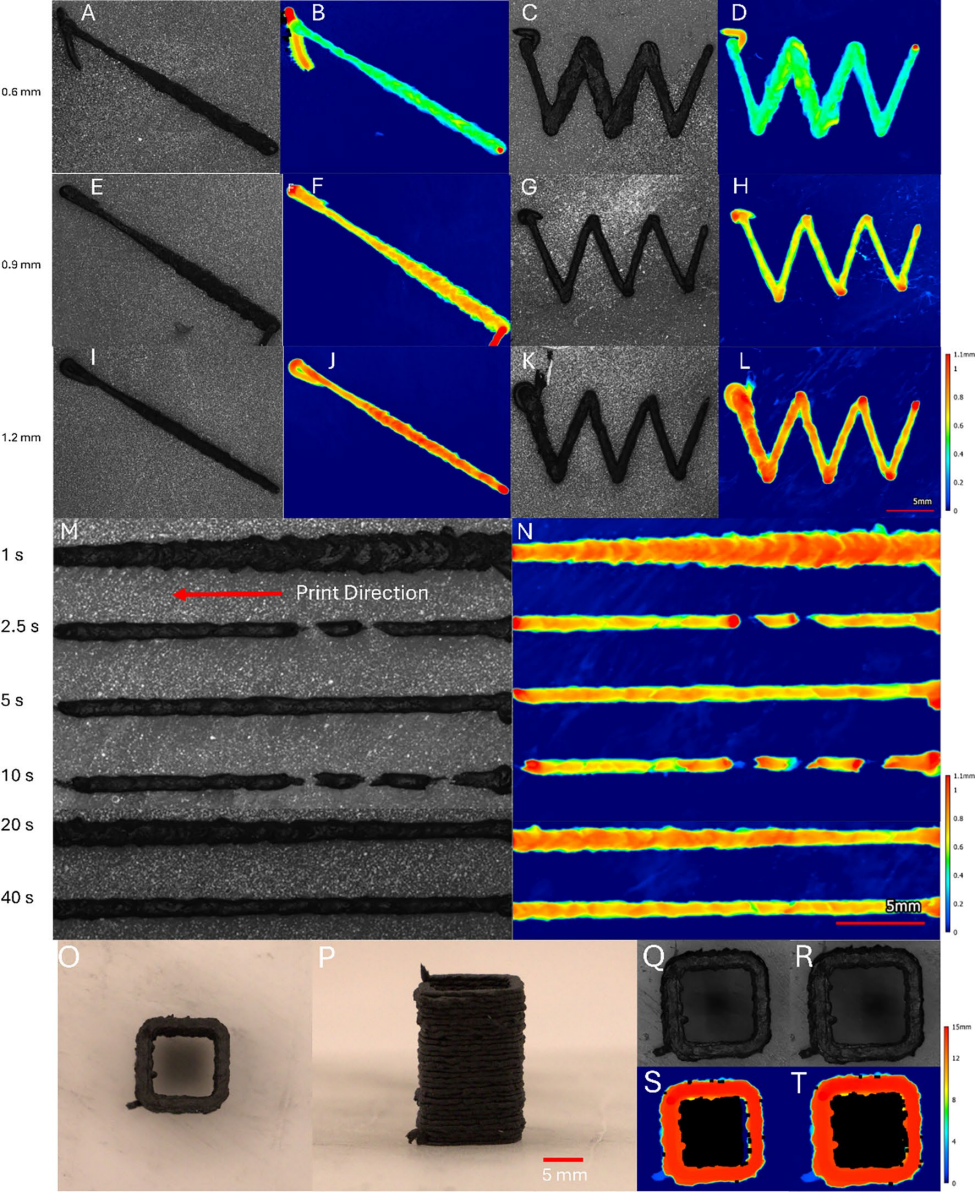
Images of line and zig-zag tests with different layer heights in each row (0.6, 0.9, and 1.2 mm). The optical images (a, e, i) and optical profilometer heat maps (b, f, j) of the line tests. The optical images (c, g, k) and optical profilometer heat maps (d, h, l) of the zig-zag tests. An optical image (m) and optical profilometer heat map (n) of the rest time test. The optical images (o, p, q, r) and optical profilometer heat maps (s, t) of a 3D-printed square single-walled tower. The images and heatmaps show the tower before (q, s) and after resting for 8 days (r, t).

Figure 2(a, b, e, f, i, j) shows the line tests, while Fig. 2(c, d, g, h, k, l) shows the zig-zag tests as a function of layer height. All tested layer height (0.6, 0.9, and 1.2 mm) lines show some variation in the bead height and width, which are visible in the profilometer heat maps. Some of this variation is inherently due to the high fiber content. The variation in the line tests, however, is more pronounced at the 0.6 layer height. The 0.6-mm layer height line has larger dips on the surface as seen in the first half of the heat map in Fig. 2(b). Despite this, there is no obvious separation of the resin from the fibers in any of the line tests. This indicates that the carbon black and fumed silica served as sufficient rheology modifiers.

From analysis of the profilometer images, all lines showed changes in line thickness throughout printing. The thickness of the bead for 0.6, 0.9, and 1.2 mm layer heights increased from 1.08 to 1.87 mm, 0.97 to 2.22 mm, and 0.97 to 1.37 mm, respectively. The bead height varies substantially both in line with and tangential to the bead as the bead thickness increases, likely the result of flow instabilities. Both the 0.6 and 0.9 mm layer heights show more pronounced flow instabilities, demonstrated by a larger change in thickness.

Zig-zag tests showed similar variations for both the 0.6- and 1.2-mm layer heights. Notably, the 0.9-mm layer height zig-zig was consistent in both bead height and width across the test. Extruding highly loaded inks is challenging because it is hard to control the extrusion pressure to avoid over- or under-extrusion. This is coupled with the fact that the *Z* height of the nozzle from the print bed will influence the pressure drop^[27]^; if the nozzle is too close, material may not extrude whereas if it is too far, geometric fidelity decreases as the material drags across a surface. Material dragging can be seen in the 0.9- and 1.2-mm zig-zag tests as evidenced by curvature in the turns. Additionally, over-extrusion is seen during turns in the profilometry scans.

Optical profilometry analysis of zig-zag tests showed a change in the bead width across all layer heights. The change was greatest among the 0.6- and 1.2-mm layer heights, increasing from 1.27 to 1.68 mm and decreasing from 1.78 to 1.08 mm, respectively. The 0.9-mm bead width decreased from 1.02 to 0.94 mm, which was the tightest range. Figures detailing the bead width measurements can be found in the Supplemental Information.

While the line tests did not show consistent bead characteristics at the 0.9-mm layer height, the zig-zag tests showed consistent beads with acceptable turning performance. Especially for a structure with turns, the 0.9-mm layer height appeared most likely to produce acceptable print performance given the other fixed parameters used in this study. Therefore, a layer height of 0.9 mm was selected to print the towers.

### Rest time test and tower print quality

Rest time tests in Fig. 2(m, n) showed significant changes in performance with different rest times. The bead width changed substantially across all tests except for tests with a rest time of 5 and 40 s. The 2.5- and 10-s rest times caused under-extrusion, while the 1- and 20-s rest times caused over-extrusion. The variety of over- and under-extrusion observed throughout the rest time tests show that print quality is influenced by stopping and starting the print, but the specific behavior varies.

After the 1- and 20-s rest times, over-extrusion and other flow instabilities become more pronounced. Particularly for the 1- and 20-s rest times, both the width and height vary periodically across the length (see Supplemental Information). The magnitude of the oscillations for the 1- and 20-s rest time tests range from approximately 0.15–0.25 mm.

The oscillatory over-extrusion is indicative of some sort of flow instability, which is likely influenced by the VAP process. It is known that the combination of high shear stress, small orifices, and highly filled polymers can induce flow instabilities, such as stick–slip, wall slip, or sharkskin.^[28,29]^ Furthermore, VAP is known for disrupting friction along the nozzle walls to enable manufacturing of viscous materials.^[13]^ However, disruption of wall friction also effectively affects the wall shear stress, which is known to contribute to wall slip.

Figure 2(o, p) shows images of a printed single-walled tower using the 60% ink. Additionally, Fig. 2(q, s) shows the initial print, while Fig. 2(r, t) shows the towers after 8 days, demonstrating the ink’s ability to hold its shape after printing. The height of the left side of the tower decreased from 14.388 to 14.330 mm (0.4% shorter), while the right side decreased from 14.621 to 14.599 mm (0.2% shorter). This extremely small deformation is supported by the high yield stress and equilibrium storage modulus measured *via* rheology.

### X-ray mCT

**Figure 3:**
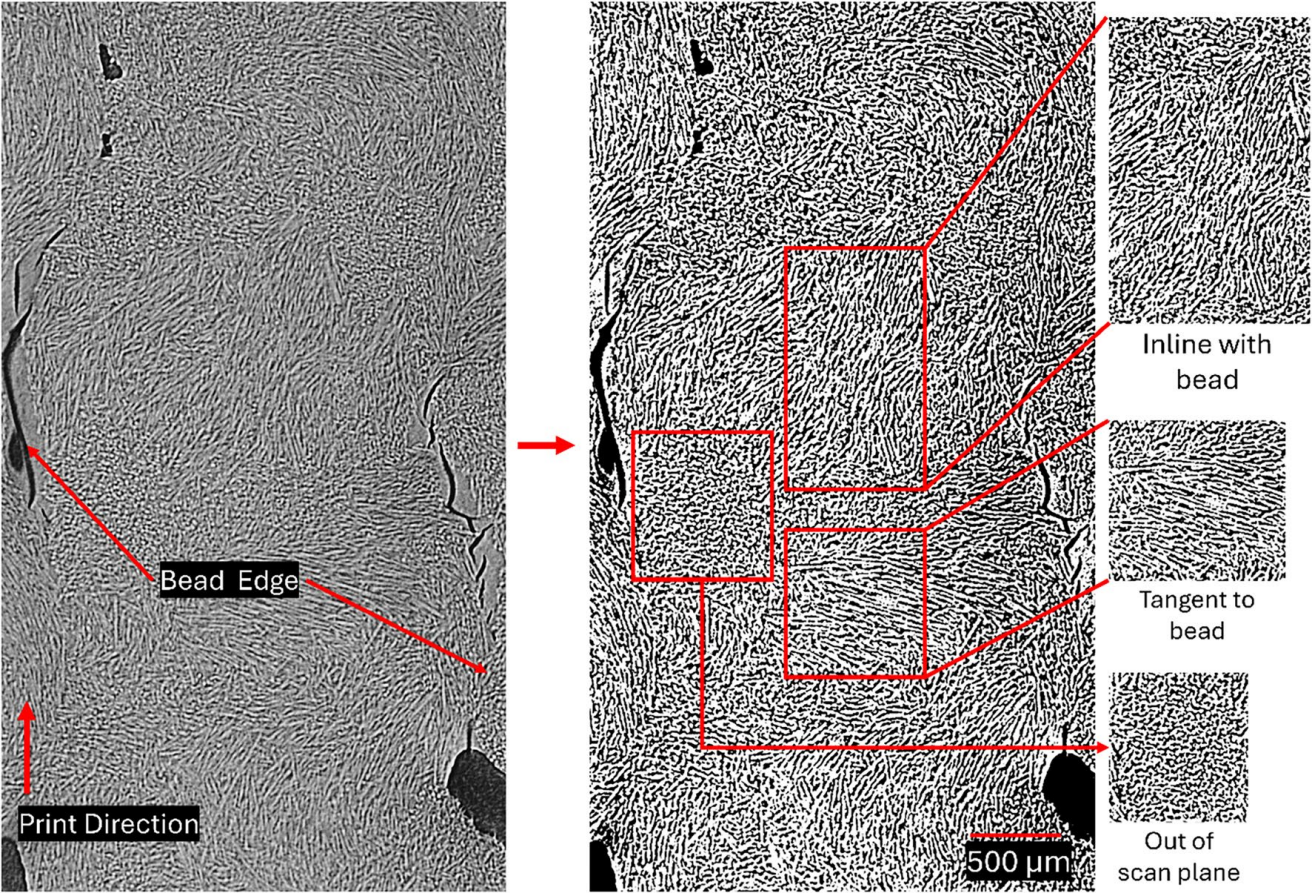
Raw (left) and threshold-filtered (right) images of a print bead under mCT. Areas with orientations inline with and tangent to the print bead can be seen. Additionally, out of scan plane orientations can be seen. Porosity and cracks are concentrated near interbead interfaces rather than within the bead.

Figure 3 shows a printed bead within the rectangular prism. In addition to visible fibers, some areas show bundles of almost perfectly round circles. These circles are likely the cross-sections of fibers oriented out of the layer plane since they have similar diameters to the other fibers, fumed silica and carbon black tend to agglomerate, and the total particle loading is < 5 wt% of the ink. The fibers appear to change orientation within < 1 cm length scales and many are not primarily oriented in the direction of printing, as is reported for other material extrusion techniques. The dominant orientation appears to shift across the bead length, changing from aligned with the print direction, to tangential to the print direction, to out of the layer plane. Because this type of orientation is not seen in material extrusion AM literature, this unique orientation behavior likely results from the vibrations which perturb the flow of the ink in a cyclic pattern. Further detail can be seen in the supplemental information.

While the lack of long-range orientation in the bead direction will decrease uniaxial strength in the print direction, the omnidirectional orientations within a bead could improve the uniformity of properties within a structure. This could be a potential advantage to obtain more uniform shrinkage during curing or pyrolysis or could provide a more torturous failure path. The orientation and direction of the fibers is likely a function of amplitude, waveform, and frequency of the vibrations. Changing the amplitude, waveform, or frequency during printing could provide a new method to control local fiber microstructure compared to other material extrusion techniques.

## Conclusion

This work outlines a combination of a viscous ceramic forming polymer resin, rheology modifiers, short fibers, and VAP to enable printing of ceramic forming green bodies with carbon fiber loadings up to 55 wt% (39.4 vol%). Rheology of the 30, 40, 50, and 60% inks were characterized to better inform their time and shear rate dependent behavior. Print quality was characterized using optical profilometry analysis of line, zig-zag, and rest time tests. The insights gained from these tests were combined to 3D print single-walled structures 15 mm tall. X-ray mCT was used to study fiber orientation.

Characterization of the rheology showed shear thinning and thixotropic behavior across all mixtures at higher shear rates. Shear thickening and rheopectic behavior were observed at low shear rates for 30, 40, and 50% mixtures, while the 60% mixture showed a high plateau in the viscosity at low shear rates. Oscillation amplitude tests showed yield stresses ranging from 0.34 to 1.65 kPa and equilibrium storage moduli from 12 to 911 kPa for 30 to 60% mixtures.

Print tests were used to characterize the print performance of the 60% mixture. The line tests showed variations in bead width across all layer heights. The zig-zag tests showed that the bead width and turning performance was the most consistent for the 0.9-mm layer height. As such, the 0.9 mm layer height was determined to have the best overall performance. The rest time experiments showed that the time-dependent nature of the ink caused over- and under-extrusion, as well as flow instabilities that caused cyclic variation in the bead profile.

Informed by rheology and printing tests, single-walled towers were printed using a continuous print path, VAP, and a layer height of 0.9 mm. Towers that were 8 days old showed insignificant shrinkage in the height (< 1%), indicating that the 60% mixture is very stable. This was also supported by the rheology results. Analysis of mCT images showed omnidirectional orientation of fibers within a printed bead with a seemly cyclic pattern. This complex fiber orientation can enable printing of parts with more isotropic properties, which can potentially be an advantage for shrinkage during processing or wear resistance. Overall, printing free standing structures with high fiber content and omnidirectional fiber alignment can be used to fabricate more complex structures in future with improved mechanical, thermal, and electrical properties depending on the application.

## Supplementary Information

Below is the link to the electronic supplementary material.

Supplementary file1 (DOCX 3178 kb)

Supplementary file2 (MP4 423727 kb)

## Data Availability

Data are available upon request.
