# Supplementary material for: Additive manufacturing and rheological characterization of ceramic matrix composite inks with high fiber volume loadings
Source: MRS Commun. 2025 Jul 31;15(4):674–81. doi: 10.1557/s43579-025-00780-3 (PMC12484376; doi:10.1557/s43579-025-00780-3)
Supplement: Supplementary file 1 — Supplementary file1 (DOCX 3178 kb) [file 43579_2025_780_MOESM1_ESM.docx]

Supplemental Information


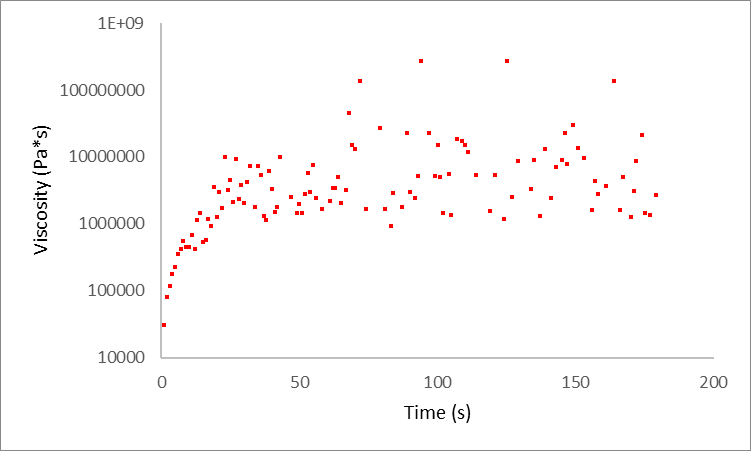


Figure S1. Viscosity of the 60% mixture as a function of time at a constant shear rate. The shear rate was maintained at 0.0001 s^-1^ for 180 seconds.

The viscosity increases during the first 30 seconds by about 2 orders of magnitude. This demonstrates rheopectic behavior which could contribute to printing difficulties. While the 60% ink only shows thixotropic behavior in the observed ramp loop data, testing with this constant shear rate over time (shown in supplemental information) shows rheopectic behavior in 60% mixtures at a shear rate of 0.0001 s^-1^.

Oscillation frequency procedures used angular velocities from 0.1 rad/s to 628 rad/s. Complex viscosities were obtained and compared to the ramp down viscosity to determine the validity of Cox-Merz rule for this system [1]. The resulting complex viscosity as a function of angular velocity can be seen in Figure S2.


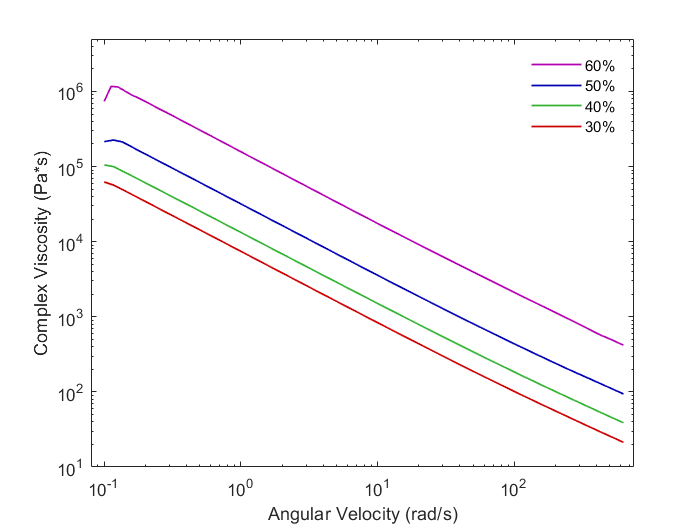


Figure S2. The complex viscosity as a function of angular velocity measured through frequency oscillation tests for the 30, 40, 50, and 60% mixtures.

The complex viscosity decreases throughout all angular velocities tested. Similar trends in complex viscosity are seen when compared to steady-state (ramp down) flow viscosity seen in Figure 1a. However the magnitude and slope of these viscosities are different, showing non-adherence to the Cox-Merz rule.


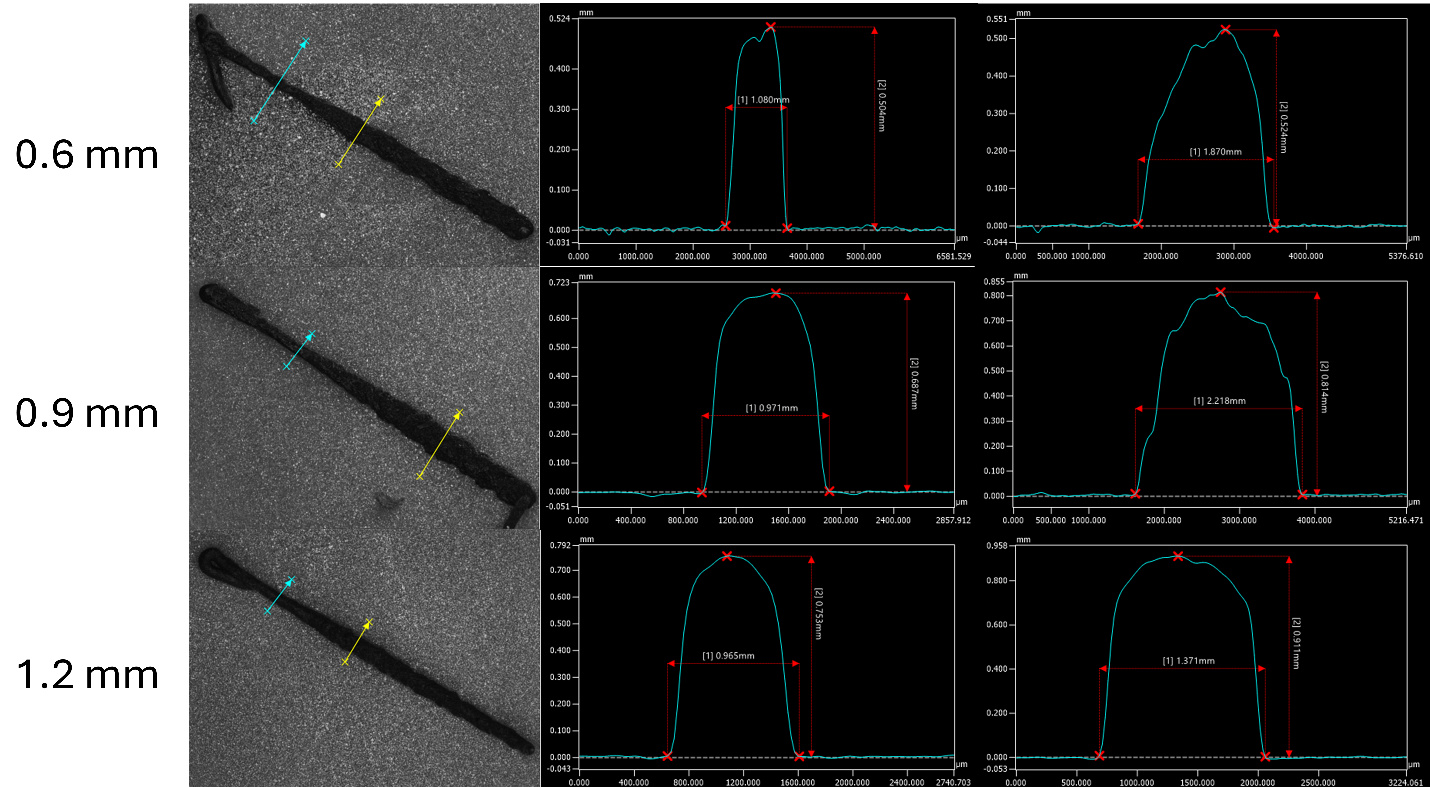


Figure S3. The optical images of the line tests as a function of layer height with the locations of the optical profilometry measurements marked in blue and yellow (left column). The measured bead profiles with the height and width are also shown (center and right columns). As the widths vary substantially between the wider and narrower sections, the height and width were measured at two points.

At the narrower points, the bead widths are similar between different layer heights, with the bead width of the 0.6 mm layer height line being slightly larger. The wider points vary from 1.37 mm for the 1.2 mm layer height, to 2.22 mm for the 0.9 mm layer height. Bead height was very similar between the wider and narrower at 0.524 mm and 0.504 mm respectively. Bead height changed more substantially for both the 0.9 and 1.2 mm layer heights, changing from 0.687 to 0.814 and 0.753 to 0.911 mm respectively.


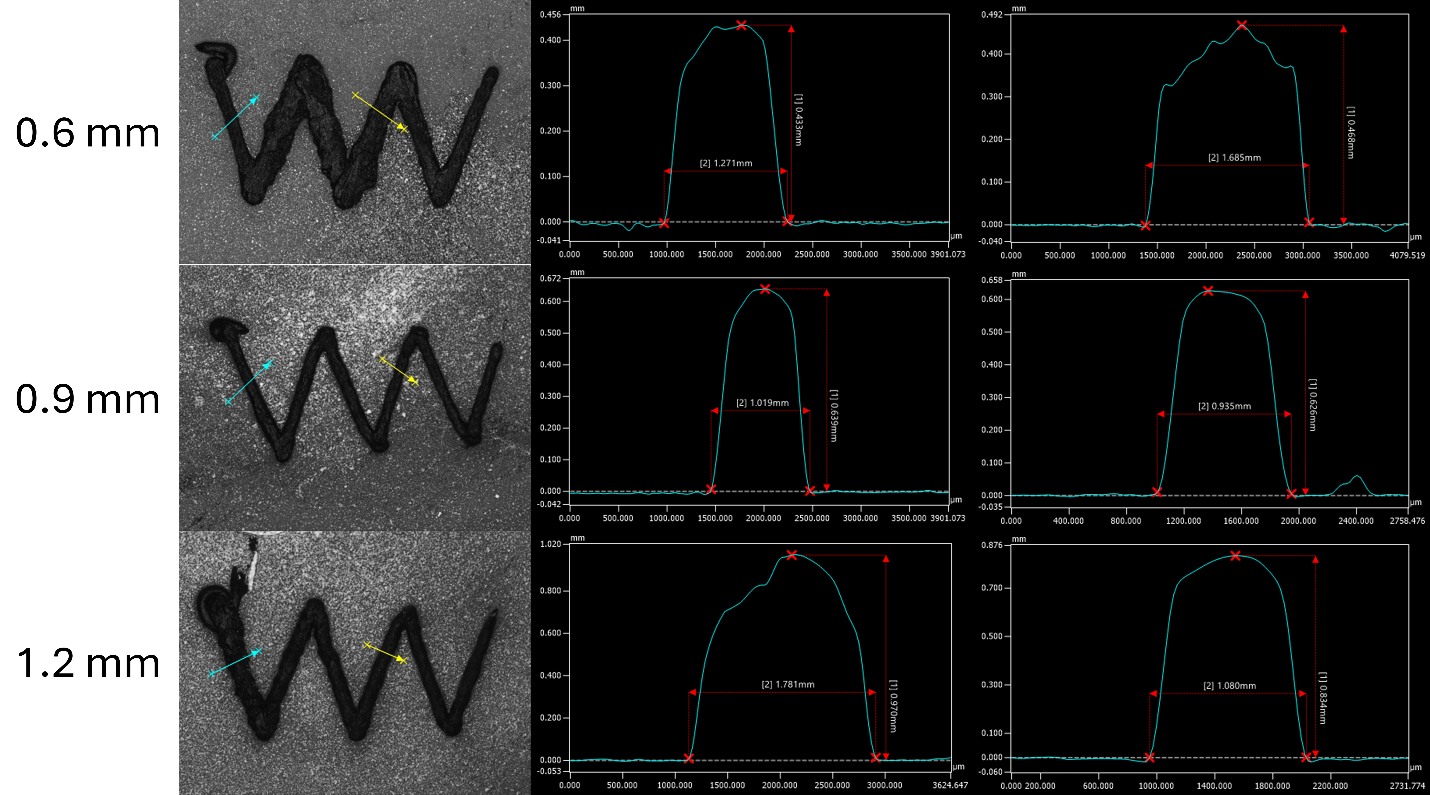


Figure S4. The optical images of the zig-zig tests as a function of layer height with the locations of the optical profilometry measurements marked in blue and yellow (left column). The measured bead profiles with the height and width at the blue mark (middle column) and the yellow mark (right column) are also shown.

Both the 0.6 and 0.9 mm tests maintained similar heights throughout the test. The bead width varied substantially across the zig-zag tests, decreasing as the print continued. The 0.9 mm offset test had the smallest decrease in bead width, while the 0.6 and 1.2 mm test both had similar changes in bead width.


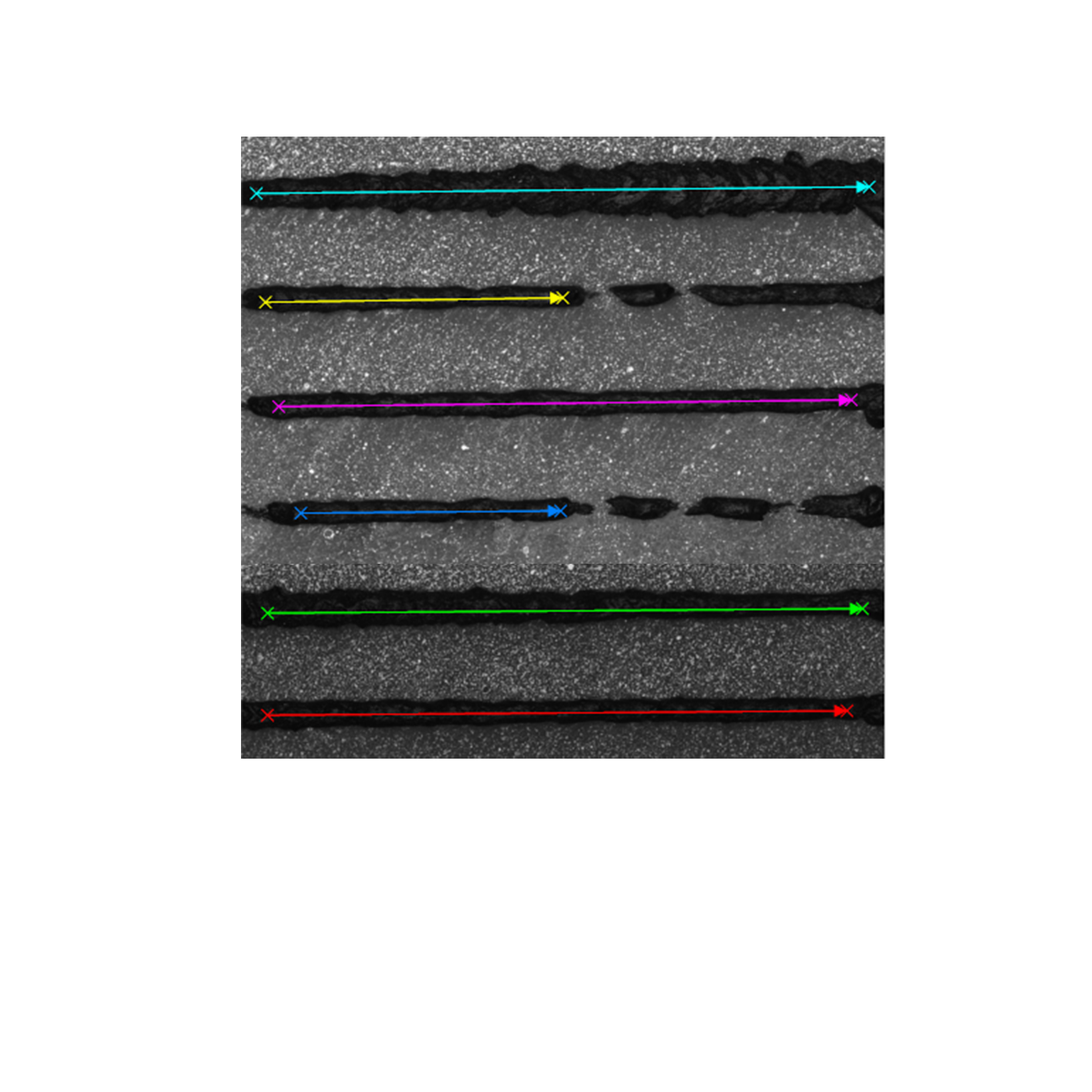


Figure S5. The optical images of the rest time tests with the locations of the optical profilometry measurements marked. The marks are labeled as follows: 1 second (light blue), 2.5 seconds (yellow), 5 seconds (purple), 10 seconds (dark blue), 20 seconds (green), and 40 seconds (orange). The profile of each line is shown in Figure S6.


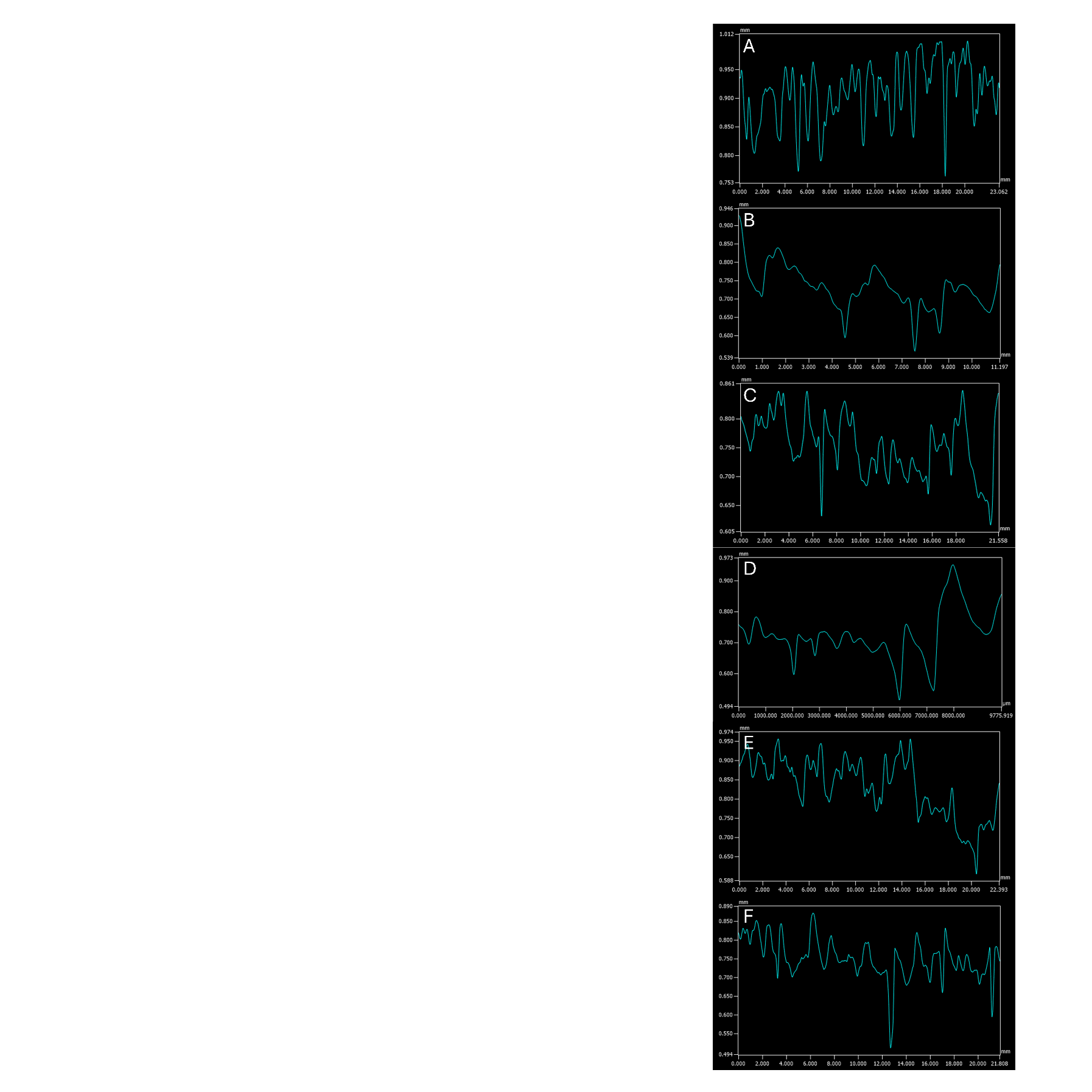


Figure S6. The measured bead profiles for rest time tests. The profiles are shown for: 1 second (A), 2.5 seconds (B), 5 seconds (C), 10 seconds (D), 20 seconds (E), and 40 seconds (F).

The magnitude and periodicity of the bead height variation is significantly more pronounced at 1, 10, 20 and 40 seconds. These variations are likely the result of flow instabilities occurring when printing with VAP.

1. Corker, A., Ng, H. C. H., Poole, R. J., and García-Tuñón, E. "3D printing with 2D colloids: designing rheology protocols to predict ‘printability’ of soft-materials," *Soft Matter* Vol. 15, No. 6, 2019, pp. 1444-1456. doi: 10.1039/C8SM01936C
